# HIV and Non‐HIV Patients Have Similar Rates of Neoplastic Findings on Screening Colonoscopy Within a Predominantly African American Cohort

**DOI:** 10.1002/cam4.70811

**Published:** 2025-04-18

**Authors:** Pooja Mude, Alexandra E. Thomson, Lavannya Atri, Samantha Newman, Carlos Palacio, John Erikson L. Yap, Christian S. Jackson, Kenneth J. Vega

**Affiliations:** ^1^ Department of Medicine Medical College of Georgia at Augusta University Augusta Georgia USA; ^2^ Department of Medicine Memorial Health University Medical Center‐HCA Savannah Georgia USA; ^3^ Department of Medicine University of Florida Health Science Center Jacksonville Florida USA; ^4^ Division of Gastroenterology & Hepatology Medical College of Georgia at Augusta University Augusta Georgia USA; ^5^ Gastroenterology Section VA Loma Linda Healthcare System Loma Linda California USA; ^6^ Division of Gastroenterology & Hepatology Prisma Health—Midlands Columbia South Carolina USA

**Keywords:** antiretroviral therapy, colorectal cancer, human immunodeficiency virus, screening colonoscopy

## Abstract

**Introduction:**

With the reduction in human immunodeficiency virus (HIV)–related mortality secondary to antiretroviral therapy, chronic medical conditions and age‐related cancers account for a larger proportion of mortality among those with HIV. Cancer risk overall remains elevated in HIV patients, and cancer screening data in this population is limited. The primary study aim was to determine whether screening colonoscopy findings differed between HIV and non‐HIV patients.

**Methods:**

A retrospective review of adults with/without HIV undergoing screening colonoscopy between February 2015 and September 2022 was performed. HIV patients were matched with non‐HIV patients by sex, race, and age, undergoing screening colonoscopy within six business days of their matched patients. Demographic data included age, race, sex, family history of colorectal cancer (CRC), smoking status, alcohol use, along with endoscopic and histologic findings that were compared between the matched pairs.

**Results:**

Ninety matched pairs of HIV and non‐HIV patients undergoing screening colonoscopy comprised the study population. The study group was 78.9% African American, 55.6% male, with a mean age of 59.0 years in HIV patients and 54.9 years in non‐HIV patients. Procedure indication was average risk screening in 91.1% of patients. No statistically significant differences in screening colonoscopy findings or polyp histology were observed between HIV and non‐HIV patients.

**Discussion:**

Similar rates of polyps were found at screening colonoscopy regardless of HIV status. CRC screening recommendations are appropriate for the HIV patient population without limitation.

## Abstract 2

The primary study aim was to determine if screening colonoscopy findings differed between HIV and non‐HIV patients. A retrospective review of adults with/without HIV undergoing screening colonoscopy between February 2015 and September 2022 was performed. Screening colonoscopy findings did not differ between patients with HIV and those without HIV in this predominantly African American study population.

## 1 Introduction

The introduction of combination antiretroviral therapy (ART) for human immunodeficiency virus (HIV) in 1996 and broad implementation of ART over the following decade have reduced HIV‐related mortality and increased life expectancy for those with HIV. Overall, HIV mortality among those on ART decreased from 17.5% to 9.5% between 1999 and 2011 [1]. Age‐related cancers and chronic medical conditions are accounting for an increasing proportion of mortality as the HIV population ages [1, 2, 3]. Antiretroviral therapy has reduced rates of non‐Hodgkin lymphoma, Kaposi sarcoma, lung, liver, and cervical cancers in those with HIV [1, 4, 5, 6]. However, people with HIV still experience a higher overall cancer risk compared to those without HIV [1, 4, 5, 7, 8]. The impact of age‐related cancers is continuing to evolve as the population of those with HIV over 50 years of age increases.

Common age‐related cancers including colorectal cancer (CRC) in those with HIV have increased in the United States over time [5, 9]. The risk of CRC in those with HIV compared to the general population is unclear in the current literature, with few studies showing those with HIV may have a reduced risk of CRC, prostate, and breast cancers [1, 5, 7]. Additionally, data regarding CRC screening rates in HIV patients is limited with substantial heterogeneity in the current literature [1, 8, 10]. It is unclear if the current CRC screening guidelines are appropriate for use in the aging HIV population [1, 5, 7, 10]. The primary aim of this study was to determine if findings on screening colonoscopy differ between HIV and non‐HIV patients.

## 2 Methods

### 2.1 Study Design

A retrospective chart review of adult patients with and without HIV undergoing screening colonoscopy at a single, tertiary academic medical center between February 2015 and September 2022. After institutional review board (IRB) approval by the Augusta University IRB, patients with HIV were identified from the Division of Infectious Disease IRB‐approved health maintenance database. Individual consent was not required by the IRB due to the retrospective nature of the study. Adult patients, 18 years or older, undergoing CRC screening colonoscopy based on indications listed by the performing endoscopist with known HIV status were eligible for inclusion. Pregnant patients, incarcerated patients, and patients undergoing diagnostic colonoscopy were excluded. HIV‐positive patients undergoing screening colonoscopy were matched with HIV‐negative patients by sex, race, age, and date of screening colonoscopy within six business days of an HIV patient. Age was matched by using age on the day of the screening procedure within plus or minus 5 years. Demographic data collected from the electronic medical record (EMR) included: age, race, sex, family history of CRC, smoking use history, alcohol use history, substance abuse history, age at HIV diagnosis, duration of HIV disease, ART medications, HIV viral load, and CD4 count. Number, size, and location of polyps or colon masses, along with endoscopy preparation quality, were collected from colonoscopy reports. Endoscopy prep quality was graded using reported quality (excellent, good, adequate, or poor) and the Boston bowel preparation scale (BBPS) [11]. The BBPS was used more commonly as it was more widely adopted during the later portion of cases included in the study. Polyp histology was obtained from procedure pathology reports and included adenomatous (tubular adenoma), hyperplastic, inflammatory, serrated adenoma, villous (tubulovillous) adenoma, and other polyp morphology.

### 2.2 Statistical Analysis

Demographic variables were analyzed using means and frequencies for nominal variables, along with mean and standard deviation (SD) for continuous variables. The HIV positive group was compared with the HIV negative group using chi‐squared/fisher exact tests for categorical variables or *t*‐tests for comparing means between the groups. A *p* value threshold of < 0.05 was used to determine statistical significance. Statistical analysis was conducted using Stata Statistical Software (version 12.1; StataCorp, College Station, Texas).

## 3 Results

A total of 90 matched pairs of HIV and non‐HIV patients who underwent screening colonoscopy comprised the study population. In 86 of the 90 pairs, this was the initial colonoscopy performed, and 82 pairs were average risk colonoscopy. Of the total 180 patients, 55.6% (*n* = 50) were male, with 78.9% (*n* = 71) African American, 18.9% (*n* = 17) non‐Hispanic White, and 2.2% (*n* = 2) Hispanic or other (Table 1). The mean age among HIV positive patients was 59.0 years, compared to 54.9 years in HIV negative patients (*p* = 0.356, Table 1). The age range was 48–71 years in the non‐HIV group and 50–75 years in the HIV group. Of the HIV positive patients, 31.1% were current smokers, compared to 35.7% of HIV negative patients (*p* = 0.641). Among the HIV study population, 91.1% were average risk screening, with 8.9% increased risk due to a family history of colon cancer. Among HIV patients, the average age at HIV diagnosis was 40.3 years, with a mean duration of HIV disease of 18.7 years. There was no statistically significant difference in demographic parameters between HIV positive and HIV negative patients (Table 1). Polyps were found in 43.3% (*n* = 39) of the HIV positive patients and 46.7% (*n* = 42) of HIV negative patients (*p* = 0.371, Table 2). More than one polyp was found in 18.9% (*n* = 17) of HIV positive patients and 21.1% (*n* = 19) of HIV negative patients. Most polyps involved the right colon, with 53.8% (*n* = 21) of those found on screening colonoscopy among HIV positive patients, compared to 59.5% (*n* = 25) among HIV negative patients. Left colon polyps accounted for 38.4% (*n* = 15) of polyps among HIV positive patients and 35.7% (*n* = 15) among non‐HIV patients (*p* = 0.102, Table 2). Rates of polyp by histology findings were similar between HIV positive and HIV negative patients (*p* = 0.505, Table 3). Adenomatous (tubular adenoma) polyps accounted for 59% of all polyps, 56% in HIV positive patients, and 62% in HIV negative patients (*p* = 0.505, Table 3). Adenoma or sessile serrated polyps were found in 23.3% (*n* = 21) of HIV positive patients and 27.8% (*n* = 25) of HIV negative patients. Most HIV patients, 85.6% (*n* = 77), were on ART, including nucleoside/nucleotide reverse transcriptase inhibitors (NRTIs) or integrase strand transfer inhibitors (INSTIs). The ART regimen included non‐nucleoside reverse transcriptase inhibitors (NNRTIs) in 20.0% of HIV patients (*n* = 18) and protease inhibitors in 13% (*n* = 13). The mean CD4 lymphocyte count was 613.1 ± 315.8 SD, and 92.2% (*n* = 83) of HIV patients had a viral load < 200 copies/mL.

**TABLE 1:** Study population demographic data.

	HIV positive, *n* = 90	HIV negative, *n* = 90	*p*
African American, *n* (%)	71 (78.9)	71 (78.9)	
Non‐Hispanic White, *n* (%)	17 (18.9)	17 (18.9)	
Hispanic/other, *n* (%)	2 (2.2)	2 (2.2)	0.721
Males, *n* (%)	50 (55.6)	50 (55.6)	1.000
Average risk, *n* (%)	82 (91.1)	80 (88.9)	0.619
Age, mean (years) ± SD	59.0 ± 5.95	54.9 ± 5.45	0.356
Current smoker, *n* (%)	28 (31.1)	30 (35.7)	0.641
Current alcohol use, *n* (%)	33 (36.7)	20 (24.1)	0.197
Average CD4 count (cells/mm^3^)	613		
Average duration HIV diagnosis (years)	18.7		

Abbreviation: HIV, human immunodeficiency virus.

**TABLE 2:** Findings on screening colonoscopy.

	HIV positive, *n* = 90	HIV negative, *n* = 90	*p*
Patients with polyps, *n* (%)	39 (43.3)	42 (46.7)	
Patients with more than 1 polyp, *n* (%)	17 (18.9)	19 (21.1)	0.371
Right colon polyp, *n* (%)	21 (53.8)	25 (59.5)	
Left colon polyp, *n* (%)	15 (38.4)	15 (35.7)	
Rectal polyp, *n* (%)	3 (7.7)	2 (4.8)	0.102

Abbreviation: HIV, human immunodeficiency virus.

**TABLE 3:** Polyp histology findings.

	HIV positive	HIV negative	Total
Adenomatous (tubular adenoma), *n* (%)	20 (55.6)	24 (61.5)	44 (58.7)
Hyperplasic, *n* (%)	7 (19.4)	8 (20.5)	15 (20.0)
Inflammatory, *n* (%)	6 (16.7)	6 (15.4)	12 (16.0)
Other, *n* (%)	2 (5.6)	0 (0.0)	2 (2.7)
Serrated adenoma, *n* (%)	1 (2.8)	0 (0.0)	1 (1.3)
Villous (tubulovillous) adenoma, *n* (%)	0 (0.0)	1 (2.6)	1 (1.3)

Abbreviation: HIV, human immunodeficiency virus.

*p* = 0.505.

## 4 Discussion

This study found similar rates of colon polyps on CRC screening colonoscopy between HIV and non‐HIV patients, with polyp detection rates of 43.3% and 46.7%, respectively, among a predominantly African American study population. There was no difference in adenomatous polyp rates of 55.6% and 61.5% between patients with HIV and without HIV, respectively. A prior retrospective cohort study of 142 adults with HIV undergoing CRC screening colonoscopy found a comparable polyp detection rate of 55% [8]. All 142 patients in the screening cohort were average risk screening for CRC cancer, with a mean age of 58 years, similar to this study [8]. Findings in this study were consistent between cohorts of predominantly average risk, regardless of HIV status. The sample size of those at increased risk due to a family history of CRC in each group prevented secondary analysis of screening findings by risk class.

The rates of risk factors for CRC, including tobacco and alcohol use, were similar between those with HIV and those without HIV in this study. The rate of tobacco use among those with HIV was 31.1% compared to 35.7% in those without HIV (*p* = 0.641). The rate of current alcohol use was 36.7% in patients with HIV and 24.1% in patients without HIV (*p* = 0.197). These findings differ from the rates reported in the current literature, which report higher rates of current and prior smoking, ranging from 56.6% to 76.0% in patients with HIV [3, 4, 7, 12]. Patients with HIV included in the current study were known to the local division of infectious disease, which educates patients on the risks of tobacco and alcohol use, which may explain the lower rates of tobacco and alcohol use among those in this study.

Although ART has reduced rates of virus‐related cancers and acquired immunodeficiency syndrome (AIDS) defining cancers in those with HIV, the overall cancer risk remains higher than that of the general population [1, 4, 5, 6, 12]. People with AIDS experience higher rates of cancers than those with HIV alone [4]. Three prior systematic reviews found a relative increase in rates of age‐related cancers, including CRC, in the setting of a significant decrease in rates of HIV and AIDS‐related cancers over the last two decades [1, 6, 9]. In the United States, two recent registry‐based studies showed that non‐AIDS—defining cancers account for increasing rates of cancer, most pronounced in older HIV patients [13, 14].

Results from prior sizeable population‐based cancer and HIV/AIDS registry studies in the United States described a decreased risk of breast cancer, prostate cancer, and CRC among the HIV population [5]. Another study looking at trends over time using data from the same combined HIV/AIDS and cancer registry found rates of CRC in HIV were increasing from 1991 through 2004, beginning to decline in 2005 [6]. Multiple studies report more advanced CRC and higher mortality in patients with HIV who develop CRC [6, 15, 16]. Systematic reviews found similar trends with higher CRC‐specific mortality among those with HIV but did not achieve statistical significance [7].

Rates of CRC screening in those with HIV vary widely in the current literature without general CRC screening recommendations modifications specific to the HIV population. A retrospective cohort study found that 25% of HIV patients at average risk for CRC cancer underwent screening colonoscopy, at a much lower frequency than general screening rates, ranging from 52% to 74% among average‐risk adults in the United States [8]. A systematic review examining CRC screening rates in those with HIV found studies reporting rates ranging from higher to lower screening rates in those with HIV compared to the general population [1]. The study identified demographic factors associated with lower screening rates in the HIV population, including younger age, fewer than 10 physician visits over a 2‐year period, lack of comorbidities, detectable viral load, and lack of family cancer history [1]. The current literature identified multiple demographic factors impacting lower screening rates for CRC, including lower socioeconomic status, inadequate insurance coverage, and competing medical needs rather than HIV alone [1, 8, 10]. This suggests demographic factors are the principal driver of screening discrepancies rather than disease‐specific factors unique to HIV. Wide ranges in rates of CRC and screening rates may reflect changes within the HIV population as more HIV patients are living longer. The reasons for this variability are believed to be multifactorial in the setting of substantial diversity within the aging HIV population.

Life expectancy for those with HIV has increased significantly over the last two decades, with improvements in disease management and ART. The difference in life expectancy between those with HIV and the general population is closing. Over 2000–2016, the difference decreased from 22.1 fewer years of life expectancy in adults with HIV to 6.8 years [2]. Adults with HIV on ART and high CD4 count achieved life expectancies near those of the general population [2]. Despite these advances in life expectancy, people with HIV experience an increased cancer‐related mortality compared to the general population, including CRC‐related mortality [16]. The magnitude of this excess mortality is greater than the sum of their respective mortality independently [17]. The increased mortality affects those with HIV of all ages. However, those with HIV and cancer over the age of 60 years had a cancer‐related mortality rate five times that of younger HIV patients with cancer [14].

In the setting of higher cancer‐related mortality in those with HIV, the importance of screening and early detection of age‐related cancers is paramount. Further understanding of factors impacting screening and CRC rates in the HIV population is crucial to ensure this population is screened effectively using all available tools to obtain the maximal benefit for these individuals. Similar findings on CRC screening in our predominantly African American study population support the use of current CRC screening guidelines in patients with HIV. The projected growth in the diverse and aging HIV population underscores the importance of continued advancement in screening, engagement, and monitoring to ensure this population is adequately screened for CRC.

## Author Contributions

**Pooja Mude:** conceptualization (equal), data curation (equal), project administration (equal), writing – original draft (equal), writing – review and editing (equal). **Alexandra E. Thomson:** data curation (equal), formal analysis (equal), writing – original draft (equal), writing – review and editing (equal). **Lavannya Atri:** data curation (equal), formal analysis (equal), writing – review and editing (equal). **Samantha Newman:** data curation (equal), formal analysis (equal), writing – review and editing (equal). **Carlos Palacio:** formal analysis (lead), methodology (lead), writing – review and editing (equal). **John Erikson L. Yap:** data curation (equal), formal analysis (equal), writing – original draft (equal), writing – review and editing (equal). **Christian S. Jackson:** formal analysis (supporting), writing – original draft (equal), writing – review and editing (equal). **Kenneth J. Vega:** conceptualization (equal), data curation (supporting), project administration (lead), supervision (lead), writing – original draft (lead), writing – review and editing (lead).

## Conflicts of Interest

The authors declare no conflicts of interest.

## Data Availability

The data that support the findings of this study are available on request from the corresponding author. The data are not publicly available due to privacy or ethical restrictions.

## References

[1] K. L. Corrigan , K. C. Wall , J. A. Bartlett , and G. Suneja , “Cancer Disparities in People With HIV: A Systematic Review of Screening for Non‐AIDS—Defining Malignancies,” Cancer 125, no. 6 (2019): 843–853, 10.1002/cncr.31838. 30645766 PMC6488220

[2] J. L. Marcus , W. A. Leyden , S. E. Alexeeff , et al., “Comparison of Overall and Comorbidity‐Free Life Expectancy Between Insured Adults With and Without HIV Infection, 2000–2016,” JAMA Network Open 3, no. 6 (2020): e207954, 10.1001/jamanetworkopen.2020.7954. 32539152 PMC7296391

[3] F. J. Palella , R. Hart , C. Armon , et al., “Non‐AIDS Comorbidity Burden Differs by Sex, Race, and Insurance Type in Aging Adults in HIV Care,” Aids 33, no. 15 (2019): 2327–2335, 10.1097/QAD.0000000000002349. 31764098 PMC12329769

[4] R. U. Hernández‐Ramírez , M. S. Shiels , R. Dubrow , and E. A. Engels , “Cancer Risk in HIV‐Infected People in the USA From 1996 to 2012: A Population‐Based, Registry‐Linkage Study,” Lancet HIV 4, no. 11 (2017): e495–e504, 10.1016/S2352-3018(17)30125-X. 28803888 PMC5669995

[5] A. E. Coghill , E. A. Engels , M. J. Schymura , P. Mahale , and M. S. Shiels , “Risk of Breast, Prostate, and Colorectal Cancer Diagnoses Among HIV‐Infected Individuals in the United States,” JNCI Journal of the National Cancer Institute 110, no. 9 (2018): 959–966, 10.1093/jnci/djy010. 29529223 PMC6136931

[6] M. S. Shiels , R. M. Pfeiffer , M. H. Gail , et al., “Cancer Burden in the HIV‐Infected Population in the United States,” JNCI Journal of the National Cancer Institute 103, no. 9 (2011): 753–762, 10.1093/jnci/djr076. 21483021 PMC3086877

[7] T. J. O'Neill , J. D. Nguemo , A. M. Tynan , A. N. Burchell , and T. Antoniou , “Risk of Colorectal Cancer and Associated Mortality in HIV: A Systematic Review and Meta‐Analysis,” JAIDS Journal of Acquired Immune Deficiency Syndromes 75, no. 4 (2017): 439–447, 10.1097/QAI.0000000000001433. 28471838 PMC5483984

[8] S. K. Nayudu , “Colorectal Cancer Screening in Human Immunodeficiency Virus Population: Are They at Average Risk?,” World Journal of Gastrointestinal Oncology 4, no. 12 (2012): 259, 10.4251/wjgo.v4.i12.259. 23443303 PMC3581851

[9] E. A. Engels , R. M. Pfeiffer , J. J. Goedert , et al., “Trends in Cancer Risk Among People With AIDS in the United States 1980–2002,” AIDS 20, no. 12 (2006): 1645–1654, 10.1097/01.aids.0000238411.75324.59. 16868446

[10] F. Momplaisir , K. Mounzer , and J. A. Long , “Preventive Cancer Screening Practices in HIV‐Positive Patients,” AIDS Care 26, no. 1 (2014): 87–94, 10.1080/09540121.2013.802276. 23742681

[11] E. J. Lai , A. H. Calderwood , G. Doros , O. K. Fix , and B. C. Jacobson , “The Boston Bowel Preparation Scale: A Valid and Reliable Instrument for Colonoscopy‐Oriented Research,” Gastrointestinal Endoscopy 69, no. 3 (2009): 620–625, 10.1016/j.gie.2008.05.057. 19136102 PMC2763922

[12] M. Helleberg , J. Gerstoft , S. Afzal , et al., “Risk of Cancer Among HIV‐Infected Individuals Compared to the Background Population: Impact of Smoking and HIV,” AIDS 28, no. 10 (2014): 1499–1508, 10.1097/QAD.0000000000000283. 24785952

[13] E. Y. Chiao , A. Coghill , D. Kizub , et al., “The Effect of Non‐AIDS‐Defining Cancers on People Living With HIV,” Lancet Oncology 22, no. 6 (2021): e240–e253, 10.1016/S1470-2045(21)00137-6. 34087151 PMC8628366

[14] M. J. Horner , M. S. Shiels , R. M. Pfeiffer , and E. A. Engels , “Deaths Attributable to Cancer in the US Human Immunodeficiency Virus Population During 2001–2015,” Clinical Infectious Diseases 72, no. 9 (2021): e224–e231, 10.1093/cid/ciaa1016. 32710777 PMC8096269

[15] M. S. Shiels , G. Copeland , M. T. Goodman , et al., “Cancer Stage at Diagnosis in Patients Infected With the Human Immunodeficiency Virus and Transplant Recipients,” Cancer 121, no. 12 (2015): 2063–2071, 10.1002/cncr.29324. 25739496 PMC4470321

[16] A. E. Coghill , M. S. Shiels , G. Suneja , and E. A. Engels , “Elevated Cancer‐Specific Mortality Among HIV‐Infected Patients in the United States,” Journal of Clinical Oncology 33, no. 21 (2015): 2376–2383, 10.1200/JCO.2014.59.5967. 26077242 PMC4500831

[17] A. E. Coghill , R. M. Pfeiffer , M. S. Shiels , and E. A. Engels , “Excess Mortality Among HIV‐Infected Individuals With Cancer in the United States,” Cancer Epidemiology, Biomarkers & Prevention 26, no. 7 (2017): 1027–1033, 10.1158/1055-9965.EPI-16-0964. PMC5500417 28619832

